# Pulmonary transit time of cardiovascular magnetic resonance perfusion scans for quantification of cardiopulmonary haemodynamics

**DOI:** 10.1093/ehjci/jead001

**Published:** 2023-01-20

**Authors:** Martin Segeroth, David Jean Winkel, Ivo Strebel, Shan Yang, Jan Gerrit van der Stouwe, Jude Formambuh, Patrick Badertscher, Joshy Cyriac, Jakob Wasserthal, Federico Caobelli, Antonio Madaffari, Pedro Lopez-Ayala, Michael Zellweger, Alexander Sauter, Christian Mueller, Jens Bremerich, Philip Haaf

**Affiliations:** Department of Radiology and Nuclear Medicine, University Hospital, Basel and University of Basel, Petersgraben 4, 4031 Basel, Switzerland; Department of Radiology and Nuclear Medicine, University Hospital, Basel and University of Basel, Petersgraben 4, 4031 Basel, Switzerland; Department of Cardiology, Cardiovascular Research Institute Basel, University Hospital Basel and University of Basel, Petersgraben 4, 4031 Basel, Switzerland; Department of Research and Analysis, University Hospital Basel, University of Basel, Petersgraben 4, 4031 Basel, Switzerland; Department of Cardiology, Cardiovascular Research Institute Basel, University Hospital Basel and University of Basel, Petersgraben 4, 4031 Basel, Switzerland; Department of Cardiology, Cardiovascular Research Institute Basel, University Hospital Basel and University of Basel, Petersgraben 4, 4031 Basel, Switzerland; Department of Cardiology, Cardiovascular Research Institute Basel, University Hospital Basel and University of Basel, Petersgraben 4, 4031 Basel, Switzerland; Department of Research and Analysis, University Hospital Basel, University of Basel, Petersgraben 4, 4031 Basel, Switzerland; Department of Research and Analysis, University Hospital Basel, University of Basel, Petersgraben 4, 4031 Basel, Switzerland; Department of Radiology and Nuclear Medicine, University Hospital, Basel and University of Basel, Petersgraben 4, 4031 Basel, Switzerland; Department of Cardiology, University Hospital Bern, Freiburgstrasse 18, 3010 Bern, Switzerland; Department of Cardiology, Cardiovascular Research Institute Basel, University Hospital Basel and University of Basel, Petersgraben 4, 4031 Basel, Switzerland; Department of Cardiology, Cardiovascular Research Institute Basel, University Hospital Basel and University of Basel, Petersgraben 4, 4031 Basel, Switzerland; Department of Radiology and Nuclear Medicine, University Hospital, Basel and University of Basel, Petersgraben 4, 4031 Basel, Switzerland; Department of Cardiology, Cardiovascular Research Institute Basel, University Hospital Basel and University of Basel, Petersgraben 4, 4031 Basel, Switzerland; Department of Radiology and Nuclear Medicine, University Hospital, Basel and University of Basel, Petersgraben 4, 4031 Basel, Switzerland; Department of Cardiology, Cardiovascular Research Institute Basel, University Hospital Basel and University of Basel, Petersgraben 4, 4031 Basel, Switzerland

**Keywords:** cardiovascular magnetic resonance imaging, pulmonary transit time, artificial intelligence, N-terminal pro-brain natriuretic peptide, heart failure, haemodynamic biomarker

## Abstract

**Aims:**

Pulmonary transit time (PTT) is the time blood takes to pass from the right ventricle to the left ventricle via pulmonary circulation. We aimed to quantify PTT in routine cardiovascular magnetic resonance imaging perfusion sequences. PTT may help in the diagnostic assessment and characterization of patients with unclear dyspnoea or heart failure (HF).

**Methods and results:**

We evaluated routine stress perfusion cardiovascular magnetic resonance scans in 352 patients, including an assessment of PTT. Eighty-six of these patients also had simultaneous quantification of N-terminal pro-brain natriuretic peptide (NTproBNP). NT-proBNP is an established blood biomarker for quantifying ventricular filling pressure in patients with presumed HF. Manually assessed PTT demonstrated low inter-rater variability with a correlation between raters >0.98. PTT was obtained automatically and correctly in 266 patients using artificial intelligence. The median PTT of 182 patients with both left and right ventricular ejection fraction >50% amounted to 6.8 s (Pulmonary transit time: 5.9–7.9 s). PTT was significantly higher in patients with reduced left ventricular ejection fraction (<40%; *P* < 0.001) and right ventricular ejection fraction (<40%; *P* < 0.0001). The area under the receiver operating characteristics curve (AUC) of PTT for exclusion of HF (NT-proBNP <125 ng/L) was 0.73 (*P* < 0.001) with a specificity of 77% and sensitivity of 70%. The AUC of PTT for the inclusion of HF (NT-proBNP >600 ng/L) was 0.70 (*P* < 0.001) with a specificity of 78% and sensitivity of 61%.

**Conclusion:**

PTT as an easily, even automatically obtainable and robust non-invasive biomarker of haemodynamics might help in the evaluation of patients with dyspnoea and HF.

## Graphical Abstract

**Graphical Abstract:**
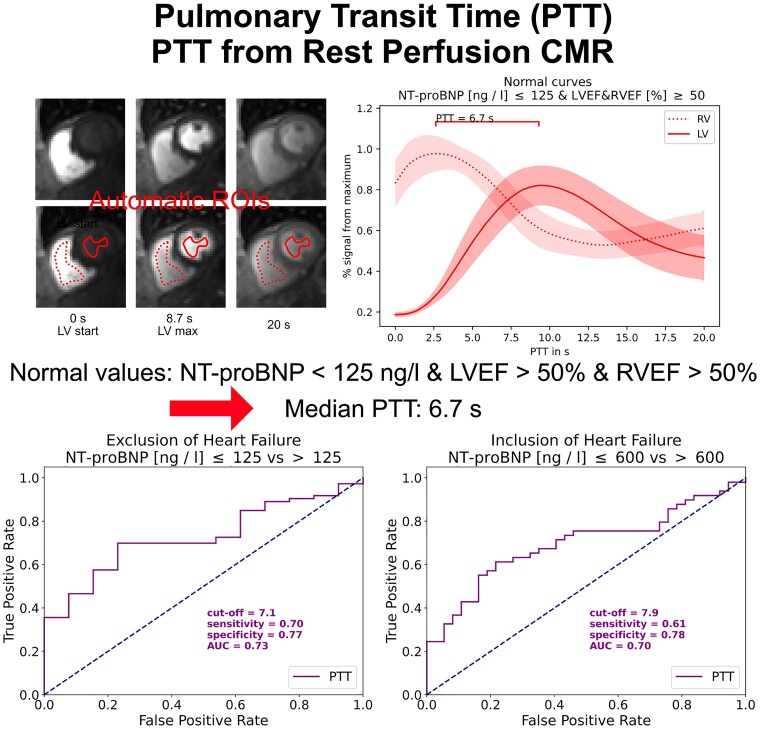

## Introduction

Heart failure (HF) is a major cause of death and disability.^1^ The prevalence of HF reaches more than 10% among people over 70 years and continued to further increase in the last decade.^1–3^

Currently, natriuretic peptides and echocardiography have a class I indication in the early diagnosis and management of HF.^1^ Natriuretic peptides such as N-terminal pro B-type natriuretic peptide (NT-proBNP) are well-established, accurate, quantitative blood biomarkers of haemodynamic cardiac stress, summarizing the presence and extent of left and right ventricular systolic and/or diastolic dysfunction as well as any valvular dysfunction.^4^ In the non-acute setting, NT-proBNP concentrations below 125 ng/L render the presence of HF very unlikely (sensitivity >95%, rule-out), while NT-proBNP concentrations above 600 ng/L increase the likelihood for HF (specificity 90%, rule-in).^4^

Unfortunately, major uncertainties remain regarding the definition and phenotyping of HF, contributing to the unacceptably high rates of mortality and morbidity.^1–3,5^ Recently, cardiovascular magnetic resonance imaging (CMR) is increasingly used in the work-up of patients with HF. We aimed to evaluate pulmonary transit time (PTT), the time it takes blood to pass from the right ventricle (RV) to the left ventricle (LV) via pulmonary circulation, and quantified PTT in CMR perfusion sequences as a novel tool to assess haemodynamics in patients with presumed HF. Furthermore, patients referred for stress perfusion CMR are often out-patients without current levels of natriuretic peptides.

PTT is affected by preload, blood volumes, global ventricular function, and pulmonary microcirculation, and it might be helpful to quantify cardiopulmonary haemodynamics.^6,7^

In this study, we aimed to investigate the clinical feasibility, inter-rater variability (agreement), and automatic assessment of PTT, as well as factors associated with normal and prolonged PTT and the association between PTT and NT-proBNP. We hypothesized that PTT has high diagnostic accuracy for HF. Furthermore, we hypothesized that PTT would also have high diagnostic accuracy for HF in the often-challenging subgroup or patients with preserved or moderately impaired left ventricular ejection fraction (LVEF).

## Methods

### Patients and study design

**Figure 1:**
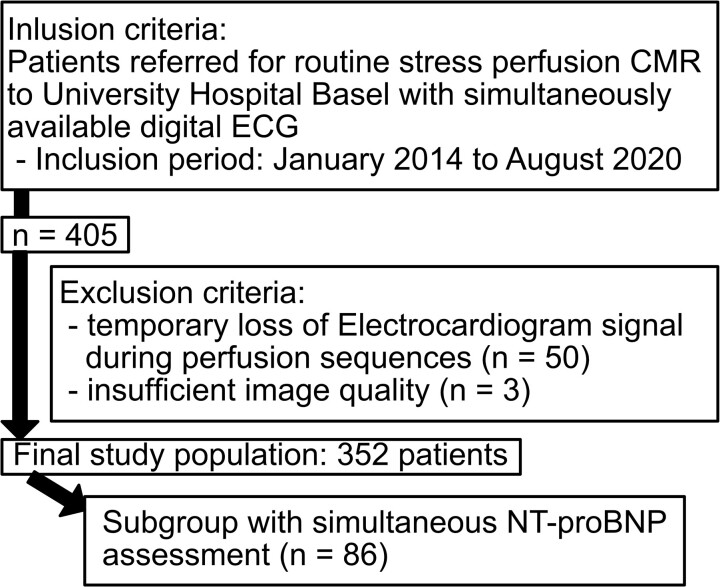
Flow chart.

All patients referred for routine stress perfusion CMR to University Hospital Basel (Switzerland), with simultaneously available digital ECGs between January 2014 and August 2020, were enrolled for this retrospective study. Of the 405 primary patients enrolled, 50 patients were excluded due to temporary loss of electrocardiogram signal during perfusion sequences, leading to time gaps during acquisition, and three patients were excluded due to insufficient image quality during acquisition. Clinical data were retrieved from the electronic patients’ records. A final diagnosis was adjudicated to each patient by at least two board-certified cardiologists and radiologists based on the CMR perfusion scan and available medical records (including patient history, electrocardiogram, results of laboratory testing, other imaging studies such as echocardiography or computed tomography and coronary angiography). Evaluation of the diagnostic accuracy of NT-proBNP is performed on the subgroup with simultaneous NT-proBNP assessment (*n* = 86) (*Figure 1*). The study was carried out according to the principles of the Declaration of Helsinki and approved by the local ethics committee.

### Biochemical analysis

Serum levels of NT-proBNP were determined with Elecsys proBNP (Roche Diagnostics, Zug, Switzerland), a quantitative electrochemiluminescence immunoassay^8^ in a dedicated core laboratory. The intra-assay coefficient of variation was 2.4% at 355 pg/mL and 1.8% at 4962 pg/mL; the inter-assay coefficients of variation were 2.9% at 355 pg/mL and 2.3% at 4962 pg/mL.^8^

### Heart failure defined by NT-proBNP

We determined the biochemical exclusion of HF by the established cutoff value of NT-proBNP level <125 ng/L and inclusion of HF for patients with NT-proBNP >600 ng/L. As recommended by current guidelines, a cutoff value >300 ng/L was chosen for the inclusion of HF for all patients with a body mass index (BMI) > 35 kg/m^2^.^1,4^

### Cardiovascular magnetic resonance: clinical protocol

CMR studies were performed on a 1.5 T or 3 T (MAGNETOM Avanto/Avanto fit resp. Skyra, Siemens Healthineers, Erlangen, Germany). A standard clinical protocol, including cine-balanced steady-state free precession imaging, adenosine stress, and rest perfusion followed by late gadolinium enhancement, was acquired for all studies with ECG-triggering. The cine sequences at 3 T had the following parameters: TE 1.47–1.61 ms; flip angle 48–63°; pixel bandwidth 780 Hz; TR 40.32–44.16 ms; spacing between slices 7.2 mm. At 1.5 T, the parameters were TE 1.1–1.15 ms; flip angle 53–74°; pixel bandwidth 930 Hz; TR 41.44–43.36 ms; spacing between slices 11.04 mm. The myocardial perfusion imaging sequence was a single-shot saturation-recovery spoiled gradient echo. Basal, midventricular, and apical short-axis perfusion images were acquired at both stress and rest. Images were acquired during 60–75 heartbeats with ECG-triggering. The temporal resolution of the perfusion scan varied with the heartbeats per minute of each patient. The perfusion sequences at 3 T had the following parameters: TE 1.03 ms; flip angle 10°; pixel bandwidth 1000 Hz; TR 158–177 ms; spacing between slices 9.6 mm. At 1.5 T the parameters were TE 1.17 ms; flip angle 12°; pixel bandwidth 1000 Hz; TR 149–174 ms; spacing between slices 9.6 mm. A bolus of 1 mmol/kg gadoterate meglumine was administered.

### Cardiovascular magnetic resonance imaging volumetric analysis and late enhancement

All CMR scans and biventricular cardiac volume parameters have been clinically assessed by joint reporting of at least two board-certified physicians (cardiologist and radiologist) with a commercially available software (SyngoVia, Siemens Healthineers, Erlangen, Germany). Biventricular volumetry was performed as proposed in the current publications.^9^ For all volumetric analyses, papillary muscles were consistently included in the ventricular blood volume.^9^ Late Gadolinium enhancement (LGE) was initially evaluated by joint reporting of at least two board-certified physicians (cardiologist and radiologist). For the purpose of this study, all LGE findings have been manually confirmed and assessed by a further reader with >10-year experience in cardiovascular imaging: LGE assessment included classification in non-ischemic vs. ischemic LGE for each of the 17 segments of the American Heart Association 17-segment.^10^ All lesions with ischemic LGE were further classified regarding their transmurality (1–25% transmurality, 25–50% transmurality, 50–75% transmurality, 75–100% transmurality).

### Cardiovascular magnetic resonance imaging pulmonary transit time

**Figure 2:**
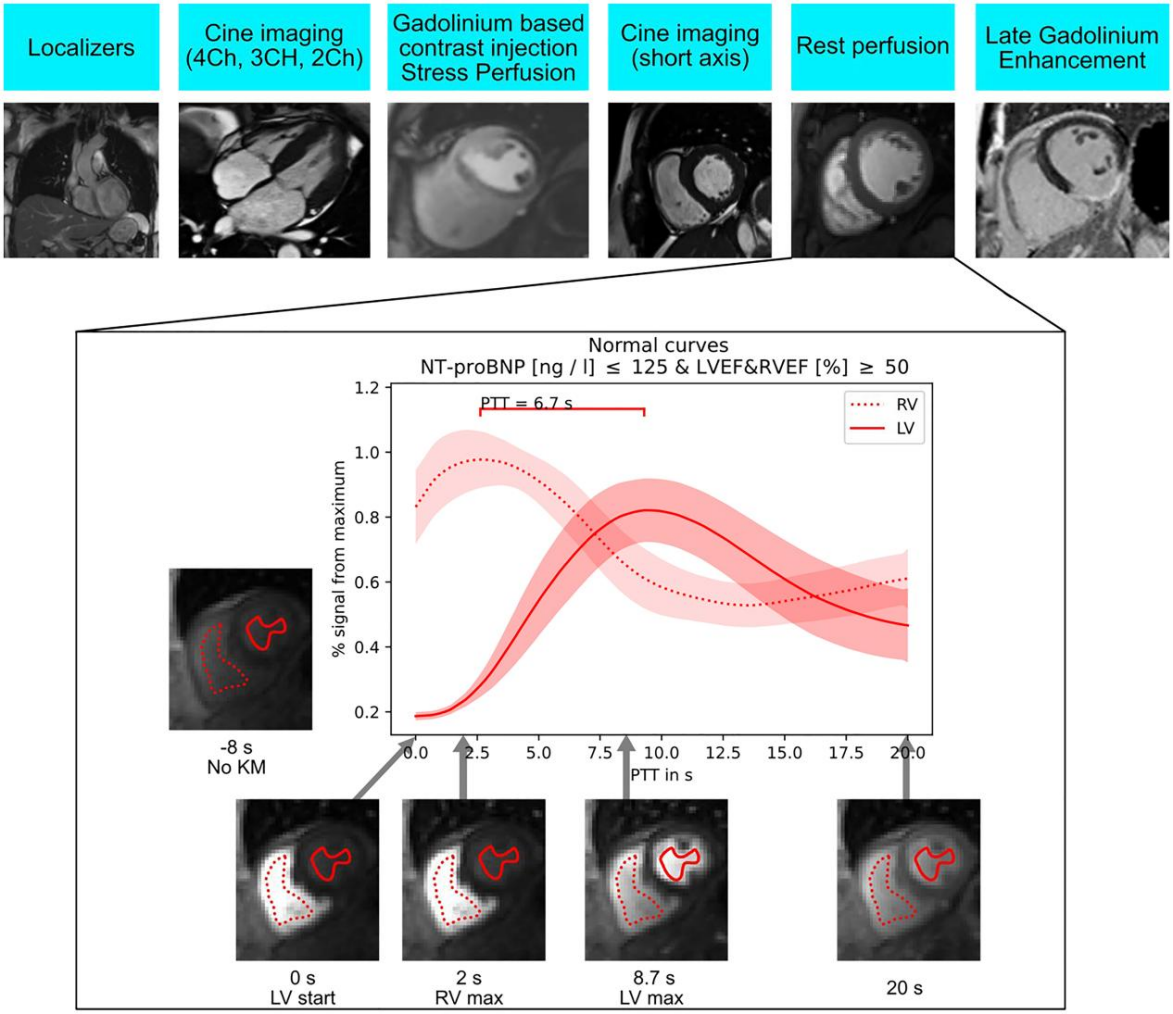
Sturdy scan protocol in the top row and PTT methods; Legend: PTT for patients with simultaneously normal values for NT-proBNP (≤125 ng/L) and both left and right ventricular ejection fraction ≥50%.

PTT was calculated from rest perfusion images with motion correction. For the determination of the PTT, region of interests (ROIs) were placed independently by three physicians in the blood pools of LV and RV sparing papillary muscles (*Figure 2*). For the analysis, Nora, a web-based framework for medical image analysis was used.^11^ Using Nora, images were viewed and readers placed ROIs in the LV and RV. The three analyzing physicians had different experience levels of CMR: Rater 1 had a quarter year of experience, rater 2 had an overall 2 years and rater 3 had more than 8 years of experience with CMR. ROIs were placed in the RV and LV for the 86 subjects who received simultaneous NT-proBNP assessment. Hereafter, a nnU-Net^12,13^ was trained based on these 86 subjects as previously described.^14^ For the evaluation of LV and RV segmentation, a dice coefficient was used. The ROI of the LV and RV were determined automatically by the deep learning-based biomedical image segmentation for the remaining subjects and all ROIs were manually evaluated by the three physicians. This evaluation was done in Nora as well using a rating: (i) correct recognition of blood pool without the inclusion of papillary muscles, (ii) correct recognition of blood pool with a minimal inclusion of papillary muscles not needing manual correction, (iii) recognition of blood pool including papillary muscles or myocardium with a need for manual correction. Three patients were excluded due to insufficient image quality. PTT was determined as the time in seconds between peak signal intensities of the time signal curves of the RV and LV, with exclusion of the recirculation component using NORA. The peak signal intensities were determined by the maxima of the time signal curves without pre-processing (such as smoothing, interpolation, or resampling). Because of its higher clinical practicability, we chose to calculate PTT with the peak-to-peak method in this analysis and not with the alternative, less frequently used, and more complicated method using ‘centers of gravity’ to determine PTT.^15^

Pulmonary transit time normalized for heart rate (nPTT) was calculated by multiplying PTT with the heart rate, equivalent to dividing PTT with the duration of the cardiac cycle (R-R interval) as used in recent studies.^16–18^ Patients’ heart rates at rest were derived from the CMR scans.

### Statistical analysis

Continuous variables were reported as means +/− SD when normally distributed and as medians [interquartile range (IQR)] otherwise. Normality was verified by a visual approach using frequency histograms and quantification using Kolmogorov–Smirnov test. Comparison between groups was performed for continuous variables using a two-tailed unpaired Student’s *t*-test or a rank-sum test depending on normality. Hypothesis testing was two-tailed. All *P*-values <0.05 were considered statistically significant. Bland–Altman plots were used for the evaluation of inter-rater variability. For evaluating the association between PTT/nPTT and NT-proBNP and between PTT/nPTT and left/right ejection fraction, scatterplots with a line of best fit were constructed. Correlations were assessed using Pearson’s correlation coefficient and Spearman’s rank correlation, respectively. The diagnostic accuracy was assessed by receiver operating characteristics curves and cutoff values were determined by the Youden-Index. Confidence intervals as well as sensitivity and specificity were estimated using bootstrapping.^19,20^ All statistical analyses were performed using Python 3.8.8.

## Results

### Baseline characteristics

Baseline characteristics for all 352 patients and their relation to PTT quartiles are illustrated in *Table 1*, relation to LVEF is illustrated in Supplementary material online, *Table S1*. The adjudicated final diagnosis was coronary artery disease in 29% of all patients, dilated cardiomyopathy in 7% of patients, acute (peri)myocarditis in 5%, and Takotsubo syndrome in 3%; 36% of patients had a normal CMR scan (*Table 1*).

**Table 1:** Baseline characteristics of all patients and relating to pulmonary transition time (nPTT) quartiles

	All patients (*n* = 353)	Q1 nPTT [<244]	Q2 nPTT [244–410]	Q3 nPTT [410–498]	Q4 nPTT [>498]	*P*-value
Demographics						
Age, years	63 (51–75)	62 (53–72)	64 (50–75)	58 (46–69)	70 (57–77)	<0.001
Sex (male), %	61%	59%	59%	59%	65%	0.95
Weight, kg	77 (65–90)	79 (66–95)	78 (69–92)	74 (61–87)	75 (63–86)	0.27
Height, m	1.72 (1.65–1.78)	1.71 (1.65–1.76)	1.72 (1.66–1.79)	1.73 (1.65–1.79)	1.72 (1.65–1.78)	0.66
Body surface area, m^2^	1.9 (1.7–2.1)	1.9 (1.8–2.1)	1.9 (1.8–2.1)	1.9 (1.7–2.1)	1.9 (1.7–2.1)	0.54
Body mass index, kg/m^2^	25.8 (22.7–29.4)	26.7 (23.2–31.4)	26.0 (23.5–29.7)	24.9 (22.1–28.3)	25.4 (22.7–28.3)	0.10
Heart rate, beats/min	59 (31–73)	30 (28–32)	35 (30–61)	67 (61–75)	77 (64–90)	<0.0001
Diabetes mellitus, %	26%	26%	23%	20%	33%	0.21
Hypercholesterolemia, %	51%	68%	38%	43%	54%	0.12
Hypertension, %	68%	76%	67%	64%	67%	0.62
History of myocardial infarction, %	68%	63%	68%	67%	73%	0.11
Aortocoronary bypass operation, %	11%	10%	7%	19%	11%	0.61
PCI, %	52%	57%	50%	48%	52%	0.30
Electrocardiogram						
Sinus rhythm, %	96%	98%	98%	100%	89%	0.87
Atrial fibrillation/atrial flutter, %	4%	2%	2%	0%	11%	<0.001
Complete left bundle branch block, %	7%	7%	9%	2%	10%	0.20
Complete right bundle branch block, %	2%	2%	2%	3%	1%	0.8
CMR volumetric parameters						
LVEF, %	54 (41–61)	58 (50–62)	53 (45–60)	55 (45–63)	39 (29–55)	<0.0001
RVEF, %	55 (49–61)	58 (52–62)	57 (49–60)	56 (51–61)	52 (38–60)	0.03
EDVI LV, mL/m^2^	87 (72–107)	82 (71–99)	90 (72–105)	85 (75–93)	103 (79–135)	<0.0001
EDVI RV, mL/m^2^	77 (66–92)	76 (65–92)	77 (66–92)	80 (71–91)	76 (64–96)	0.85
SV LV, ml	83 (68–99)	88 (70–100)	85 (70–102)	86 (74–99)	72 (61–89)	<0.001
SV RV, ml	80 (66–96)	86 (66–103)	82 (68–94)	84 (70–99)	73 (58–85)	<0.001
Myocardial mass indexed, g/m^2^	66 (57–83)	63 (55–74)	66 (55–80)	64 (59–74)	81 (66–99)	<0.0001
CMR late gadolinium enhancement (LGE)						
Myocardial Infarction (ischemic LGE pattern)	19%	18%	17%	11%	28%	0.07
Non-ischemic fibrosis (non-ischemic LGE pattern)	21%	14%	26%	17%	26%	0.16
Final adjudicated diagnosis						
Coronary artery disease, %	29%	31%	25%	25%	35%	0.53
Dilated cardiomyopathy, %	7%	6%	3%	3%	16%	<0.005
Acute (peri-) myocarditis, %	5%	1%	7%	8%	6%	0.22
Takotsubo syndrome, %	3%	0%	2%	1%	7%	0.026
Hypertrophic Cardiomyopathy, %	3%	2%	5%	0%	5%	0.22
Other cardiomyopathy, %	4%	5%	5%	6%	1%	0.46
Cardiac Sarcoidosis/amyloidosis, %	1%	0%	0%	1%	2%	0.30
Normal CMR Scan, %	36%	50%	35%	43%	15%	<0.001
Unclear diagnosis, %	10%	6%	14%	10%	9%	0.40
Other, %	3%	0%	5%	2%	5%	0.22

Values are displayed as median [interquartile range] or %.

CMR, cardiovascular magnetic resonance; LVEF, left ventricular ejection fraction; RVEF, right ventricular ejection fraction; EDVI, end-diastolic volume indexed; LV, left ventricle; RV, right ventricle; PCI, percutaneous coronary intervention; Information about comorbidities were available for 58% of patients. Continuous variables with a significant group comparison are itemized in Supplementary material online, *Figure S1*.

Patients with higher PTT values were older and presented more frequently with atrial fibrillation/flutter, higher left-ventricular end-diastolic volumes/masses, and lower ejection fractions. Patients with higher PTT values more often had dilated cardiomyopathy and Takotsubo syndrome and, less frequently, a normal CMR scan.

### Inter-rater variability of pulmonary transit time

**Figure 3:**
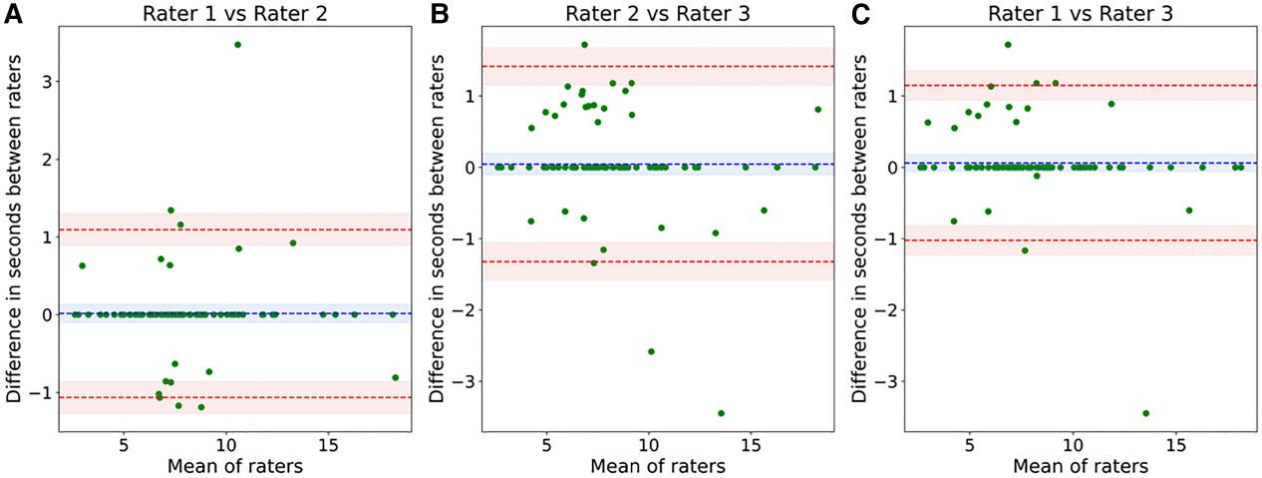
Bland–Altman plots for PTT between rater 1 and rater 2 (*A*), rater 2 and rater 3 (*B*), rater 1 and rater 3 (*C*).

PTT has been obtained manually by means of placing ROIs in < 1 min per patient for the 86 patients who received simultaneous NT-proBNP assessment. The median average PTT over all readers was 7.5 s (IQR: 6.3—9.0 s) and the median average nPTT amounted to 549 (IQR: 420–735). The median PTT for rater 1 amounted to 7.6 s (IQR 6.3–8.9 s), for rater 2–7.7 s (IQR: 6.4–9.0 s), and for rater 3–7.5 s (IQR 6.3–8.8 s). The median difference of all three raters regarding the PTT amounted to 0.0 s (IQR: 0.0–0.0 s) with a significant correlation of rater 1 and 2 of 0.98 (*P* < 0.0001), rater 2 and rater 3 of 0.98 (*P* < 0.0001), as well as rater 1 and rater 3 of 0.98 (*P* < 0.0001) (*Figure 3*). As the nPTT is the PTT corrected by the heart frequency, it showed similar correlations.

### Pulmonary transit time obtained by deep learning-based biomedical image segmentation

Automatic placement of ROIs in the right and LVs was trained on the 86 patients, where all readers placed ROIs in the right and LVs. As the nnU-Net was trained on the ROIs of reader 1, the dice-score between the nnU-Net and reader 1 is 1 for the LV and RV. The dice between the nnU-Net and reader 2 or reader 3 is lower but not below the dice between reader 1 and reader 2 or reader 3 (see Supplementary material online, *Figure S2*). PTT was obtained automatically and correctly in all 266 patients, as assessed independently by three raters (77% with ROIs completely excluding papillary muscles and 23% with minimal inclusion of papillary muscles without the need of manual correction as assessed by the three independent raters). None of the ROIs included papillary muscles of the myocardium, needing manual correction (exemplary ROIs placed by nnU-Net see Supplementary material online, *Figure S3*).

### Pulmonary transit time in patients with biventricular ejection fraction >50% and normal NT-proBNP values

A total of 182 patients had both left and right ventricular ejection fraction above 50% (LVEF median 60%, IQR 55–65%; RVEF 60%, IQR 56–63%). Their median PTT values amounted to 6.8 s (IQR 5.9–7.9 s) and their median nPTT values amounted to 370 (IQR 220–448). Of the 86 patients who received simultaneous NT-proBNP assessment, 10 patients simultaneously had NT-proBNP values below 125 ng/L (median 57 ng/L, IQR 13–84 ng/L) and both left and right ventricular ejection fraction above 50% (LVEF median 63%, IQR 58–67%; RVEF median 60%, IQR 59–64%). Their PTT values amount to 6.7 s (CI 5.4–7.7 s) (*Figure 2*).

### Pulmonary transit time according to levels of the left and right ventricular ejection fraction

**Figure 4:**
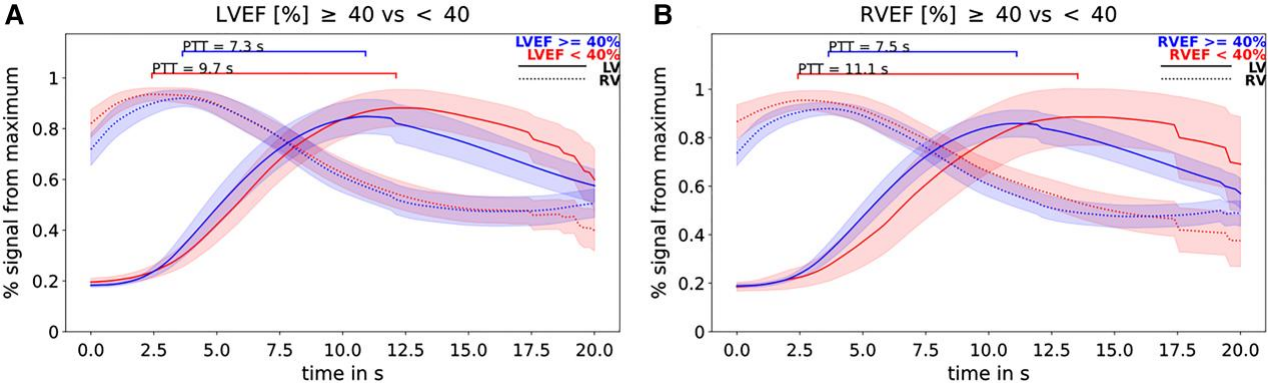
PTT and LVEF/RVEF; legend: (*A*) lower values of pulmonary transit time of patients with preserved LVEF (≥40%; *n* = 263) compared with patients with reduced LVEF (<40%; *n* = 78). (*B*) Lower values of the pulmonary transit time of patients with preserved RVEF (≥40%; *n* = 305) compared with patients with reduced RVEF (<40%; *n* = 34).

The median LVEF amounted to 54% (IQR: 41–61%) and the median RVEF amounted to 55% (IQR: 49–61%). Patients with reduced LVEF (<40%) demonstrate significantly higher values of PTT than patients with preserved LVEF (≥40%) (9.3 s vs. 7.1 s; *P* < 0.001) (*Figure 4A*). Similarly, PTT is significantly higher in patients with reduced RVEF (<40%) than in patients with preserved RVEF (≥40%) (9.3 s vs. 7.3 s; *P* = 0.0001) (*Figure 4B*). Values of PTT and nPTT correlate significantly with both LVEF (see Supplementary material online, *Figure S4*) and RVEF (see Supplementary material online, *Figure S5*).

### Pulmonary transit time for evaluation of heart failure as assessed by NT-proBNP

**Figure 5:**
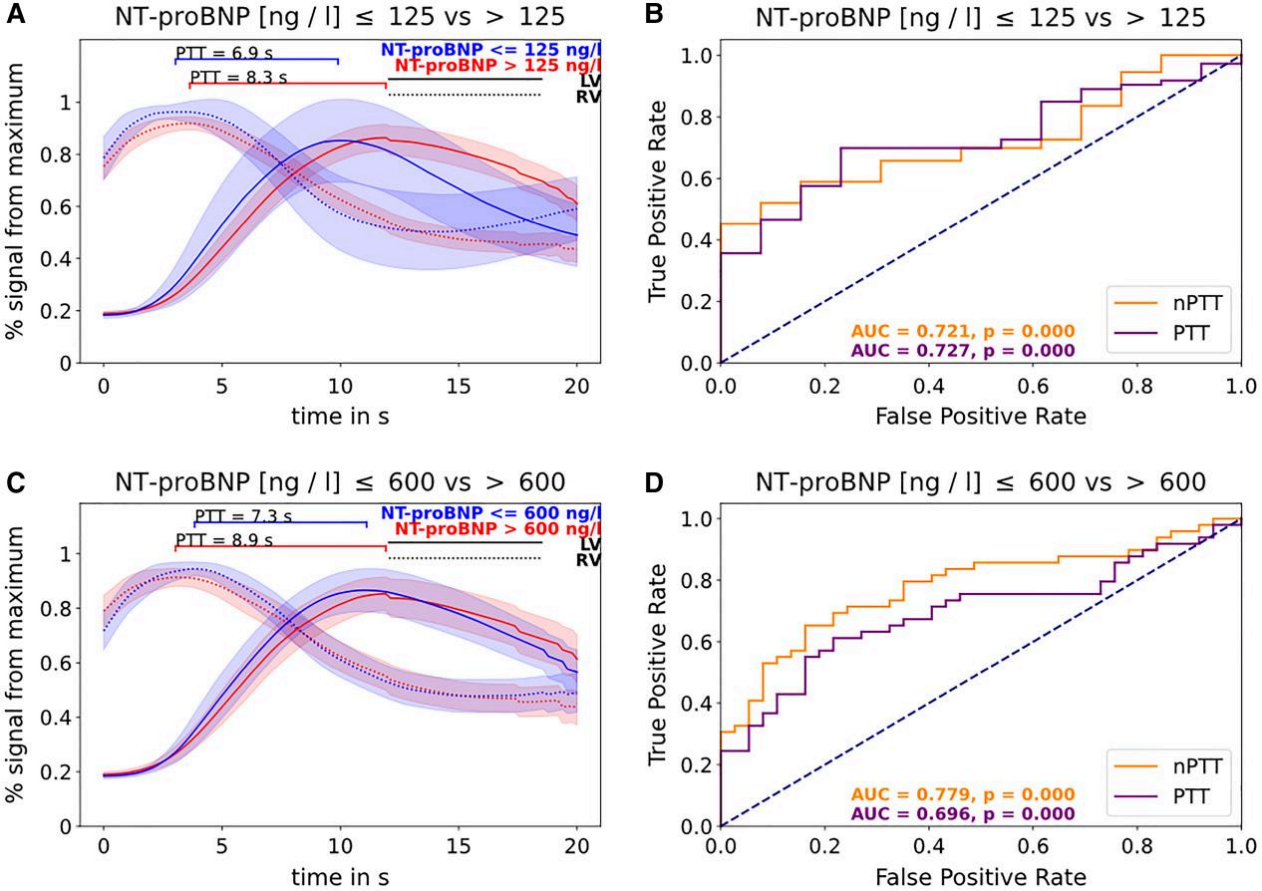
PTT according to NT-proBNP inclusion and exclusion of heart failure; legend: (*A*) lower values of pulmonary transit times of patients with NT-proBNP ≤ 125 ng/L compared with patients with NT-proBNP > 125 ng/L; (*B*) diagnostic performance of PTT and nPTT in receiver operating characteristic (ROC) curve analysis for the exclusion of heart failure as defined by NT-proBNP values ≤125 ng/L; (*C*) values of pulmonary transit times of patients with NT-proBNP ≤ and > 600 ng/L, respectively, with NT-proBNP ≤ and > 300 ng/L for patients with BMI > 35 kg/m^2^; (*D*) diagnostic performance of PTT and nPTT in receiver operating characteristic (ROC) curve analysis for the inclusion of heart failure as defined by NT-proBNP values >600 ng/L. In patients with BMI > 35 kg/m^2^, a NT-proBNP ≤ and > 300 ng/L was used for the inclusion of heart failure.

The median time between CMR and NT-proBNP acquisition was 30.8 h (IQR: 20.0–46.7 h). Median NT-proBNP amounted to 881 ng/L (IQR: 314–2844 ng/L). NT-proBNP correlated significantly with nPTT (see Supplementary material online, *Figure S6*). The diagnostic accuracy of PTT and nPTT for the exclusion of HF (NT-proBNP < 125 ng/L) as quantified by the area under the ROC curve (AUC) was 0.73 (CI 0.59–0.84; *P* < 0.001) for PTT and 0.72 (CI 0.59–0.83; *P* < 0.001) for nPTT. A PTT < 7.1 s resulted in a specificity of 77% and a sensitivity of 70% to exclude HF. A nPTT < 634 resulted in a specificity of 100% and a sensitivity of 45% to exclude HF (*Figures 5*A and B).

For the inclusion of HF (NT-proBNP > 600 ng/L for BMI ≤35 kg/m^2^ and > 300 ng/L for BMI > 35 kg/m^2^), the diagnostic performance of PTT amounted to an AUC of 0.70 (CI 0.58–0.80; *P* < 0.001), and for nPTT, to an AUC of 0.78 (CI 0.68–0.87; *P* < 0.001), whereas a PTT > 7.8 s for the inclusion of HF resulted in a specificity of 78% and sensitivity of 61%. A nPTT > 586 for the inclusion of HF resulted in a specificity of 84% and sensitivity of 65% (*Figures 5*C and D).

### Pulmonary transit time for the inclusion of heart failure as assessed by NT-proBNP in patients with preserved or moderately impaired ventricular ejection fraction

For patients with a LVEF greater than or equal to 30% (≥30%; *n* = 65) the diagnostic performance of PTT for the inclusion of HF [NT-proBNP > 600 ng/L (>300 ng/L if BMI > 35 kg/m^2^)] amounts to an AUC of 0.62 (CI 0.48–0.76, *P* = 0.05) and for nPTT to an AUC of 0.77 (CI 0.65–0.89, *P* < 0.001) with a specificity of 58% and a sensitivity of 69% for a PTT > 7.1 s, and a specificity of 70% and a sensitivity of 75% for a nPTT > 475 (see Supplementary material online, *Figures S7A* and *B*).

For the subpopulation with a RVEF greater than or equal to 30% (≥30%; *n* = 74) the diagnostic performance of PTT for inclusion of HF amounted to an AUC of 0.66 (CI 0.53–0.77, *P* = 0.007), and for nPTT, to an AUC of 0.75 (CI 0.64–0.86, *P* < 0.001) with a specificity of 77% and a sensitivity of 56% for a PTT > 7.9 s and a specificity of 89% and a sensitivity of 56% for a nPTT > 586 (see Supplementary material online, *Figures S7C* and *D*).

For the subpopulation with both LVEF and RVEF greater than or equal to 30% (*n* = 62), the diagnostic performance of PTT for the inclusion of HF amounted to an AUC of 0.62 (CI: 0.48–0.76, *P* = 0.05), and for nPTT, to an AUC of 0.75 (CI: 0.60–0.87, *P* < 0.001) with a specificity of 61% and a sensitivity of 69% for a PTT > 7.1 s, and a specificity of 70% and a sensitivity of 72% for a nPTT > 475 (see Supplementary material online, *Figures S7E* and *F*).

## Discussion

In this retrospective study of patients referred for routine stress perfusion CMR, we scrutinized the accuracy of PTT and nPTT for robust quantification of cardiopulmonary haemodynamics and benefit in the assessment of HF. We report 4 major findings with the potential to improve diagnosis in patients with unclear dyspnoea and HF:

First, PTT and nPTT are rapidly and easily obtainable, robust, non-invasive biomarkers of haemodynamics that can be obtained automatically by routine perfusion CMR with very low inter-rater variability. Secondly, in this study, patients with biventricular function >50% and normal NT-proBNP values presented with the lowest PTT values of 6–8 s. These values are similar to asymptomatic controls of other studies and might represent the normal range of PTT.^21^ Thirdly, both reduced left and right ventricular ejection fractions were associated with a prolonged PTT and nPTT. Fourth, the diagnostic accuracy of PTT and nPTT for both exclusion and inclusion of HF, as assessed by NT-proBNP, was moderate to high, even in patients with only moderately impaired or preserved left or right ventricular ejection fraction.

PTT represents the time it takes for a bolus of intravenous contrast to pass from the right to the LV. The clinical utility of PTT has first been examined by an invasive dye-dilution method and simultaneous invasive bilateral cardiac catheterization in 1960.^22^ Initially, PTT could only be derived invasively by right and left heart catheterization and was demonstrated to correlate with the New York Heart Association functional classification in patients with mitral stenosis,^23^ as well as HF and pulmonary hypertension.^24^ Non-invasive methods to derive PTT from CMR ^16,21,25^ have later been deployed. Additional methods to derive the PTT from computed tomography ^17^ and echocardiography ^7^ have also been explored. Recently, it has been demonstrated that PTT can be derived automatically from CMR data by deep learning and is, therefore, easy to obtain;^18^ we confirm this finding. Since PTT is calculated from rest perfusion images, no pharmacologic stress agents are necessary. Furthermore, the acquisition of rest first-pass perfusion images during the application of a gadolinium-based contrast agent will not lead to a relevant prolongation of the CMR scan. By using a deep learning-based biomedical image segmentation, PTT might even be automatically and reliably available as demonstrated in this study.

PTT has been demonstrated to be altered in various diseases, including HF,^26,27^ pulmonary hypertension,^28,29^ chronic lung disease,^30^ and to correlate with cardiac function.^17^ Similarly, elevated levels of NT-proBNP are a marker of haemodynamic stress and NT-proBNP may be elevated due to various factors such as volume overload and ventricular or valvular dysfunction. Therefore, factors leading to an increased PTT and elevated NT-proBNP seem to be overlapping considerably.

To the best of our knowledge, this is the first CMR study to assess and compare the diagnostic accuracy of PTT for the presence or absence of HF as assessed by NT-proBNP. Of note, in this analysis, we do not assess HF itself, but compare two non-invasive biomarkers with each other. Both natriuretic peptides and PTT are unspecific, non-invasive biomarkers to quantify cardiopulmonary haemodynamic stress, and may be elevated in varying degrees due to the aforementioned factors. PTT might also be elevated in patients with altered pulmonary micro- or macro-circulation such as chronic lung disease.^30^ NT-proBNP is known to be an independent predictor of respiratory exacerbations in COPD patients.^31^

Our study extends and corroborates earlier contrast-enhanced ultrasound ^32^ and CMR studies analyzing the association between prolonged PTT and dilated LV, reduced LV systolic function, and HF in patients with low cardiac-output.^16,26^ Cao et al. ^16^ assessed global circulation transit time (TT) from the right atrium to ascending aorta, in patients with HF with reduced (HFrEF) and preserved LVEF (HFpEF). TT was more prolonged in patients with HFrEF than in patients with HFpEF, while normal controls had the lowest TTs. Our data confirm these findings as the PTT for the subpopulation with reduced LVEF (<40%) was significantly longer than in patients with a LVEF ≥40%. Furthermore, we demonstrated in our analysis that in the subpopulation with LVEF and RVEF ≥30%, it is possible to identify patients with an abnormal high NT-proBNP with high diagnostic accuracy.

Backhaus et al. ^33^ demonstrated that real time-CMR allows highly accurate identification of HFpEF during physiologic exercise and qualifies as a suitable non-invasive diagnostic alternative to right heart catheterization. Sine physiologic exercise during real time-CMR is elaborate and not well established in clinical practice, PTT might serve as a screening tool.

In our study, patients with HFpEF or HFrEF with normal NT-proBNP values were classified as normal, due to their normal NT-proBNP, but their prognosis is associated with their NT-proBNP levels.^34^ Even though patients with HFpEF might also present with normal NT-proBNP levels, it was shown that irrespective of the presence of HFpEF or HFrEF, discharge NT-proBNP levels predicted outcome and mortality similarly.^35^ In out-patients referred for CMR examinations, NT-proBNP is often not available. Therefore, a prolonged PTT might induce the assessment of NT-proBNP with a further clinical investigation and improve patient outcome.

The diagnostic utility of natriuretic peptides in patients with unclear dyspnoea and presumed HF has been extensively demonstrated and natriuretic peptides are widely used in clinical practice,^1,4^ for the inclusion and exclusion of HF and the differential diagnosis of cardiac vs. pulmonary dyspnoea. Patients referred for stress perfusion CMR are often out-patients without current laboratory assessments including current levels of natriuretic peptides. Therefore, the automatic quantification of PTT might be helpful as a tool to guide further diagnostics or render HF, unlikely in patients with normal PTT values and otherwise normal CMR studies.

In our analysis, a slightly better discrimination in terms of the inclusion and exclusion of HF was observed for nPTT (PTT normalized for the heart rate). This effect might, in part, be explainable by an influence of heart rate on NT-proBNP levels.^36^ For this analysis, we chose to calculate and report both PTT and nPTT to allow optimal comparison with the few previous studies on this subject. PTT as the time it takes blood to pass from the right to the LV via pulmonary circulation may be understood more intuitively than nPTT, and can be calculated very easily. Therefore, PTT may have a higher clinical potential than nPTT, a dimensionless number. On the other hand, PTT as a non-invasive marker of global cardiopulmonary haemodynamics is affected by many variables, such as preload, blood volume, global ventricular function, or pulmonary microcirculation. A normalization of PTT for heart rate corrects for this important factor influencing PTT and may lead to a better comparability of PTT between patients, and also longitudinally in the same patient.

Limitations of this study were as follows: First, as a retrospective study, we cannot quantify exactly the clinical benefit of PTT for the inclusion and exclusion of HF (as determined by NT-proBNP). Second, patients with HFpEF and HFrEF are only classified as HF if their NT-proBNP is abnormal. Third, the sample size is limited in this study since the majority of patients referred for CMR perfusion scans were out-patients without simultaneously available NT-proBNP values. This may have led to a selection bias since only certain patients will get an NT-proBNP assessment. This may also explain the relatively low number of patients with normal NT-proBNP values in our population. Fourth, although patients with insufficient image quality or obvious temporary loss of ECG signal during perfusion sequences were excluded from the analysis, we cannot guarantee that single ECG signals have been lost or inadequately detected during perfusion sequences, which might have had an influence on the accuracy of PTT. Furthermore, we only assessed patients with dyspnoea and/or suspected coronary artery disease.

To conclude PTT as a non-invasive marker of cardiopulmonary haemodynamics can be easily, even automatically and robustly obtained in routine CMR scans. It may help in the diagnostic assessment and characterization of patients with unclear dyspnoea or HF.

## Supplementary material

Supplementary materials are available at the *European Heart Journal - Cardiovascular Imaging* online.

## Supplementary Material

jead001_Supplementary_Data

## Data Availability

The data underlying this article will be shared on reasonable request to the corresponding author.
