# Supplementary material for: Pulmonary transit time of cardiovascular magnetic resonance perfusion scans for quantification of cardiopulmonary haemodynamics
Source: Eur Heart J Cardiovasc Imaging. 2023 Jan 20;24(8):1062–71. doi: 10.1093/ehjci/jead001 (PMC10364617; doi:10.1093/ehjci/jead001)
Supplement: jead001_Supplementary_Data [file jead001_supplementary_data.docx]

**Supplementary files**

**Maschine Learning Checklist:**

| 1 | Designing the Study Plan |
| --- | --- |
| 1.1 | Describe the need for the application of machine learning to the dataset  Automatic detection of the right and left ventricle for determination of PTT in 269 patients, clinical application and further research |
| 1.2 | Describe the objectives of the machine learning analysis  Automatic placement of ROIs in the right and left ventricle |
| 1.3 | Define the study plan  Manual placement of ROIs in the right and left ventricle in 86 Patients.  Training an nnU-Net.  Prediction of ROIs in 269 Patients and evaluation by 3 raters. |
| 1.4 | Describe the summary statistics of baseline data  CMR Images of the basis of the heart |
| 1.5 | Describe the overall steps of the machine learning workflow  Page 9 |
| 2 | Data Standardization, Feature Engineering, and Learning |
| 2.1 | Describe how the data were processed in order to make it clean, uniform, and consistent  nnU-Net worklflow |
| 2.2 | Describe whether variables were normalized and if so, how this was done  Default normalization by nnU-Net |
| 2.3 | Provide details on the fraction of missing values (if any) and imputation methods  Page 9 |
| 2.4 | Describe any feature selection processes applied  NA |
| 2.5 | Identify and describe the process to handle outliers if any  NA |
| 2.6 | Describe whether class imbalance existed, and which method was applied to deal with it  NA |
| 3 | Selection of Machine Learning Models |
| 3.1 | Explicitly define the goal of the analysis e.g., regression, classification, clustering  Automatic segmentation of the right and left ventricle for determination of PTT |
| 3.2 | Identify the proper learning method used (e.g., supervised, reinforcement learning etc.) to address the problem  Supervised |
| 3.3 | Provide explicit details on the use of simpler, complex, or ensemble models  Default workflow of nnU-Net |
| 3.4 | Provide the comparison of complex models against simpler models if possible  There is no alternative for this task |
| 3.5 | Define ensemble methods, if used  We trained the 5 models with FOLD 5 cross validiation. The images would be 5 time predicted with these 5 models. The final results were the average of 5 predictions. |
| 3.6 | Provide details on whether the model is interpretable  Can place ROIs in the right and left ventricle. The ROIs were be evaluated by 3 raters. |
| 4 | Model Assessment |
| 4.1 | Provide a clear description of data used for training, validation, and testing  Manual placement of ROIs in the right and left ventricle in 86 Patients served as training dataset. The training was validated using cross validation. Testing and further validation was performed by prediction of ROIs in 269 Patients and evaluation by 3 raters. |
| 4.2 | Describe how the model parameters were optimized (e.g., optimization technique, number of model parameters etc.)  nnU-Net workflow |
| 5 | Model Evaluation |
| 5.1 | Provide the metric(s) used to evaluate the performance of the model  Dice-score (see nnU-Net), Cross validation, mean dice of LV is 0.80, RV 0.76. Detection rate is for LV 1, RV is 1. |
| 5.2 | Define the prevalence of disease and the choice of the scoring rule used  NA |
| 5.3 | Report any methods used to balance the numbers of subjects in each class  NA |
| 5.4 | Discuss the risk associated to misclassification  With not perfect segmentation it could lead fat gradient of perfusion signal. Normally there is not influence for detecting the peaks. Only in case of total false ROI placement, it leads to false PTT, which was checked by 3 raters or radiologist by clinical application. |
| 6 | Best Practices for Model Replicability |
| 6.1 | Consider sharing code or scripts on a public repository with appropriate copyright protection steps for further development and non-commercial use  nnU-Net -> already free  Trained nnU-Net model |
| 6.2 | Release a data dictionary with appropriate explanation of the variables  NA |
| 6.3 | Document the version of all software and external libraries used  nnU-Net |
| 7 | Reporting Limitations, Biases and Alternatives |
| 7.1 | Identify and report the relevant model assumptions and findings  Image presented contains the heart with the right and left ventricle |
| 7.2 | If well performing models were tested on a hold-out validation dataset, detail the data of that validation set with the same rigor as that of training dataset (see section 2 above)  Testing and further validation was performed by prediction of ROIs in 269 Patients and evaluation by 3 raters. |

*Supplement Table 1*: Baseline Characteristics of all patients and relating to LVEF

|  | LVEF  [<30%]  (n=34) | LVEF  [30 – 40%]  (n=44) | LVEF  [40 – 55%]  (n=121) | LVEF  [> 55%]  (n=142) | p-value |
| --- | --- | --- | --- | --- | --- |
| Demographics | | | | | |
| Age, years | 68 (61 – 77) | 68 (57 – 78) | 63 (50 – 74) | 61 (50 – 73) | 0.0039 |
| Sex (male),% | 69% | 71% | 66% | 49% | 0.15 |
| Weight, kg | 77 (68 – 95) | 81 (65 – 93) | 79 (68 – 94) | 73 (62 – 85) | 0.026 |
| Height, m | 1.71 (1.66 – 1.8) | 1.74 (1.68 - 1.78) | 1.74 (1.67 – 1.81) | 1.69 (1.63 – 1.76) | 0.008 |
| Body surface area, m^2^ | 1.9 (1.8 - 2.1) | 1.9 (1.8 – 2.1) | 1.9 (1.8 – 2.1) | 1.8 (1.7 – 2.0) | 0.011 |
| Body mass index, kg/m^2^ | 27.0 (23.1 – 30.5) | 27.4 (23.4 – 29.8) | 25.8 (23.2 – 29.4) | 24.9 (22.0 – 28.5) | 0.21 |
| Heart rate, beats / min | 74 (48 – 84) | 68 (35 – 87) | 57 (31 – 69) | 54 (31 – 71) | < 0.0001 |
| Diabetes mellitus,% | 39% | 43% | 26% | 13% | < 0.0001 |
| Hypercholesterolemia,% | 32% | 57% | 51% | 57% | 0.27 |
| Hypertension,% | 71% | 80% | 67% | 63% | 0.0225 |
| History of myocardial infarction,% | 70% | 75% | 81% | 57% | 0.0033 |
| Aortocoronary bypass operation,% | 15% | 4% | 4% | 20% | 0.15 |
| PCI,% | 50% | 64% | 54% | 49% | 0.0049 |
| Electrocardiogram | | | | | |
| Sinus rhythm,% | 95% | 98% | 95% | 97% | 0.996 |
| Atrial fibrillation/Atrial flutter,% | 5% | 2% | 5% | 3% | 0.65 |
| Complete left bundle branch block,% | 26% | 9% | 6% | 2% | < 0.0001 |
| Complete right bundle branch block,% | 0% | 0% | 0% | 4% | 0.076 |
| CMR volumetric parameters | | | | | |
| LVEF,% | 24 (20 – 25) | 35 (32 – 36) | 51 (45 – 54) | 62 (60 – 66) | < 0.0001 |
| RVEF,% | 40 (29 – 50) | 52 (42 – 60) | 54 (48 – 58) | 60 (55 – 64) | < 0.0001 |
| EDVI LV, ml/m^2^ | 151 (124 – 171) | 111 (96 – 129) | 87 (75 – 100) | 76 (68 – 87) | < 0.0001 |
| EDVI RV, ml/m^2^ | 88 (67 – 101) | 72 (63 – 87) | 80 (69 – 94) | 76 (65 – 87) | 0.28 |
| SV LV, ml | 63 (53 – 74) | 76 (60 – 89) | 85 (69 – 99) | 87 (72 – 105) | < 0.0001 |
| SV RV, ml | 64 (49 – 79) | 74 (58 – 87) | 84 (67 – 98) | 85 (69 – 101) | < 0.0001 |
| Myocardial mass indexed, g/m^2^ | 102 (89 – 116) | 81 (69 – 99) | 67 (58 – 79) | 60 (52 – 69) | < 0.0001 |
| CMR Late Gadolinium Enhancement (LGE) | | | | | |
| Myocardial Infarction (ischaemic LGE pattern) | 31% | 44% | 15% | 11% | < 0.0001 |
| Non-ischaemic Fibrosis (non-ischaemic LGE pattern) | 33% | 27% | 20% | 14% | 0.0742 |
| Final adjudicated diagnosis | | | | | |
| Coronary artery disease,% | 41% | 49% | 26% | 24% | 0.0216 |
| Dilated cardiomyopathy,% | 31% | 16% | 6% | 0% | < 0.0001 |
| Acute (Peri-) Myocarditis,% | 0% | 2% | 6% | 6% | 0.33 |
| Takotsubo Syndrome,% | 0% | 7% | 3% | 2% | 0.24 |
| Hypertrophic Cardiomyopathy,% | 0% | 2% | 3% | 4% | 0.66 |
| Other Cardiomyopathy,% | 5% | 2% | 5% | 3% | 0.78 |
| Cardiac Sarcoidosis / Amyloidosis,% | 0% | 2% | 0% | 0% | 0.41 |
| Normal CMR Scan,% | 0% | 4% | 35% | 53% | < 0.0001 |
| Unclear diagnosis,% | 18% | 11% | 12% | 6% | 0.10 |
| Other,% | 5% | 4% | 4% | 1% | 0.40 |

Values are displayed as median [interquartile range] or %.

CMR = Cardiovascular Magnetic Resonance, LVEF = Left ventricular ejection fraction, RVEF = Right ventricular ejection fraction, EDVI = end diastolic volume indexed, LV = left ventricle, RV = right ventricle, PCI = Percutaneous coronary intervention; Information about comorbidities were available for 58% of patients.


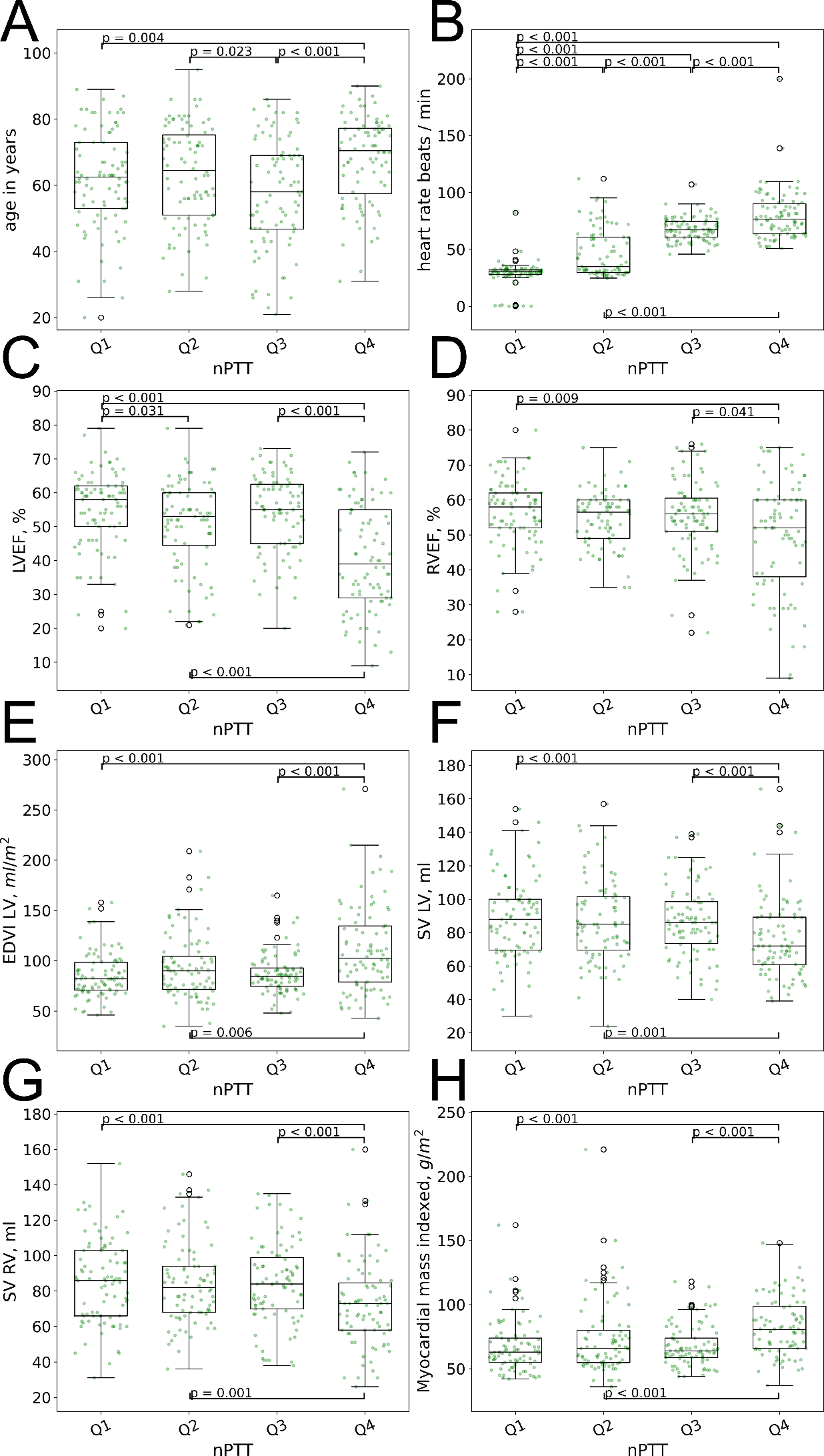


*Supplement Figure 1S*: Groups with a significant finding in Table 1. A) age in years, B) heart rate, C) LVEF , D) RVEF, E) EDVI LV, F) SV LV, G) SV RV, H) myocardial mass index according to quartiles of nPTT.


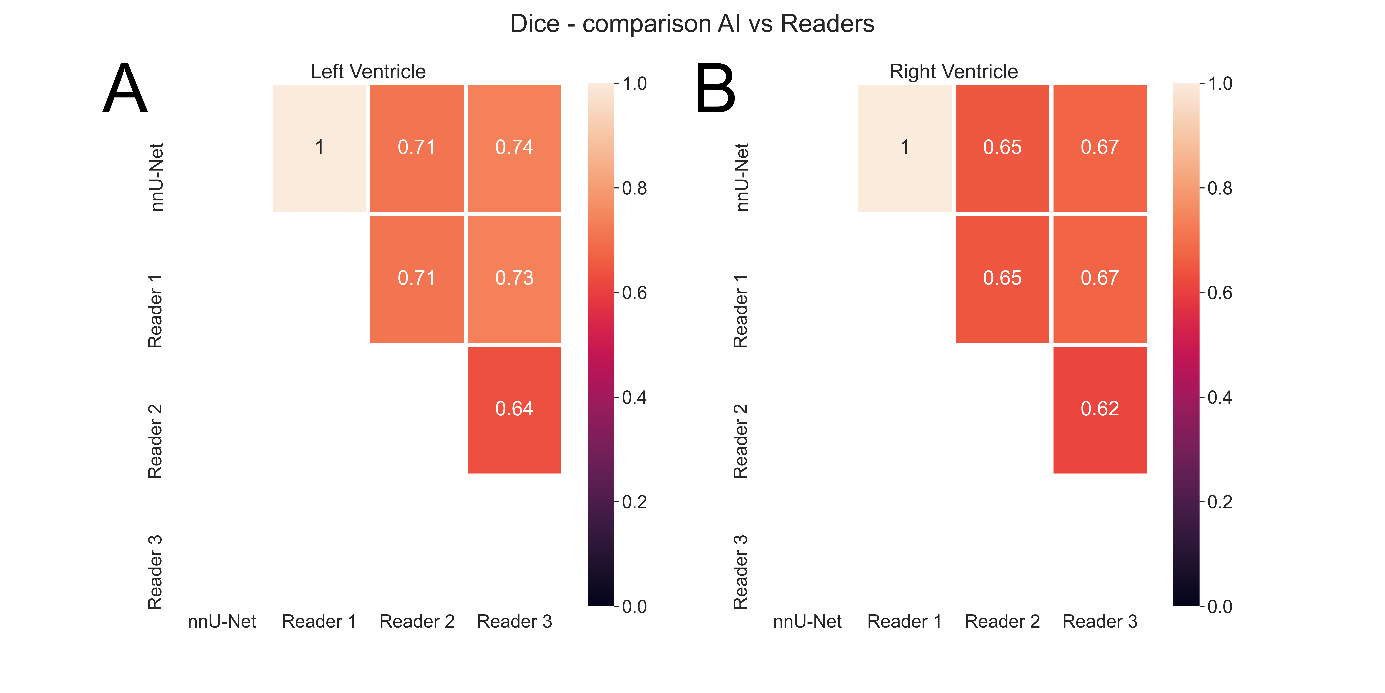


*Supplement Figure 2S*: Dice-score for comparison nnU-Net and all readers for ROIs in the A) left ventricle and B) right ventricle.


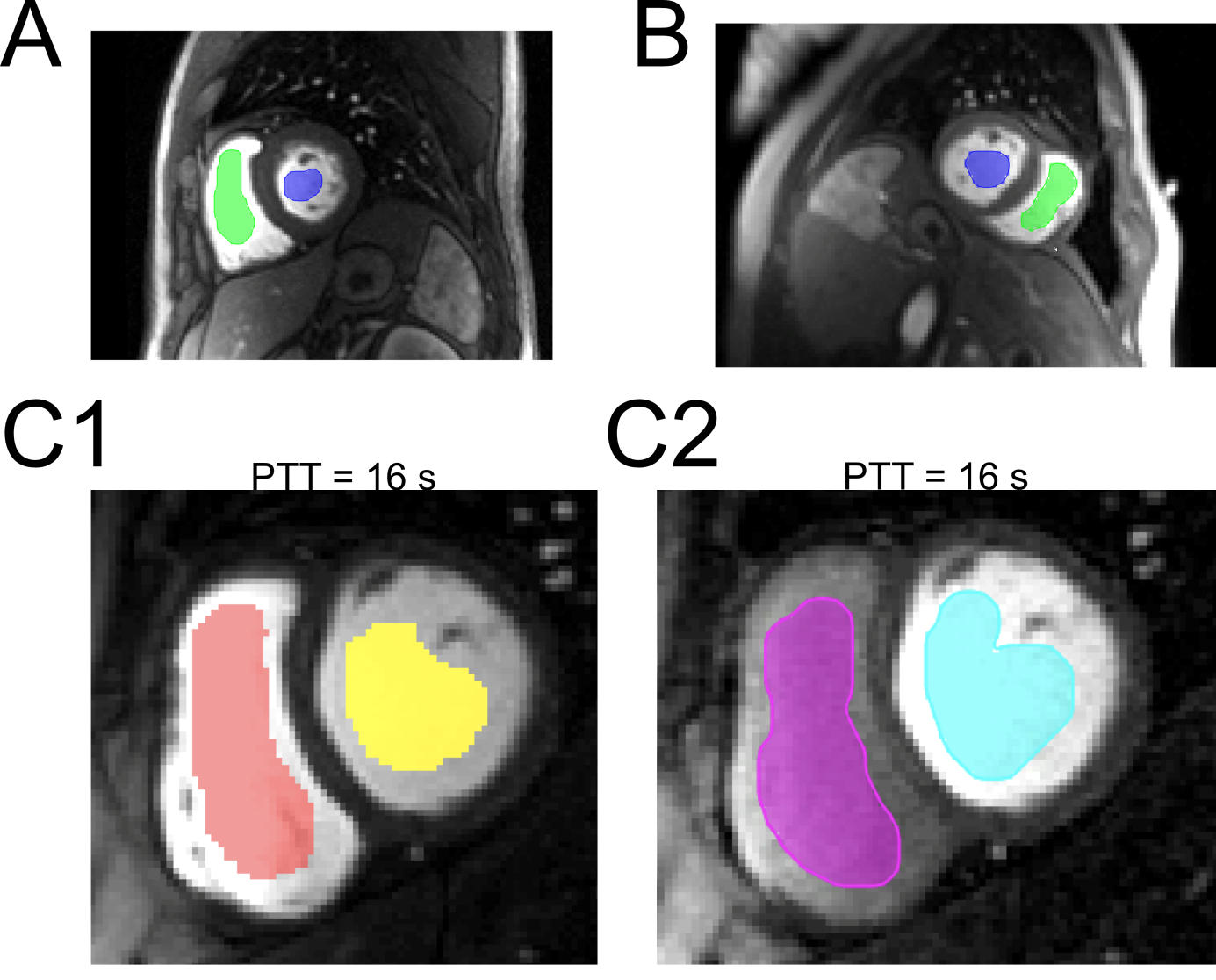


*Supplement Figure 3S*: Exemplary ROI placement by nnU-Net in the right (green) and left (blue) ventricle for 2 patients (A,B). C1 exemplary ROI placement by nnU-Net in the right (red) and left (yellow) ventricle for cross validation in comparison to reader 3 (right ventricle: purple; left ventricle light blue) showing same PTT.


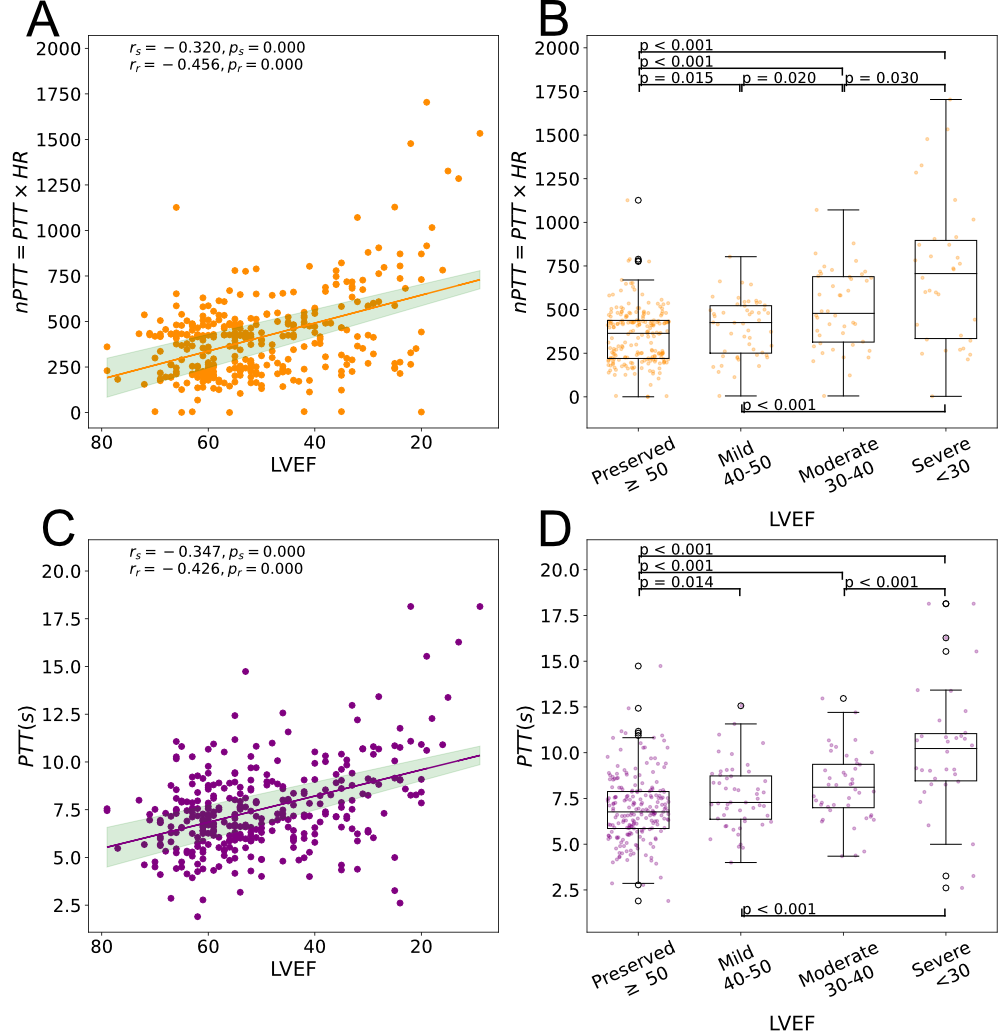


*Supplement Figure 4S*: A) Correlation between normalized pulmonary transition time (nPTT) and left ventricular ejection fraction (LVEF); B) nPTT according to groups of LVEF; C) Correlation between pulmonary transit time (PTT) and LVEF; D) PTT according to groups of LVEF.


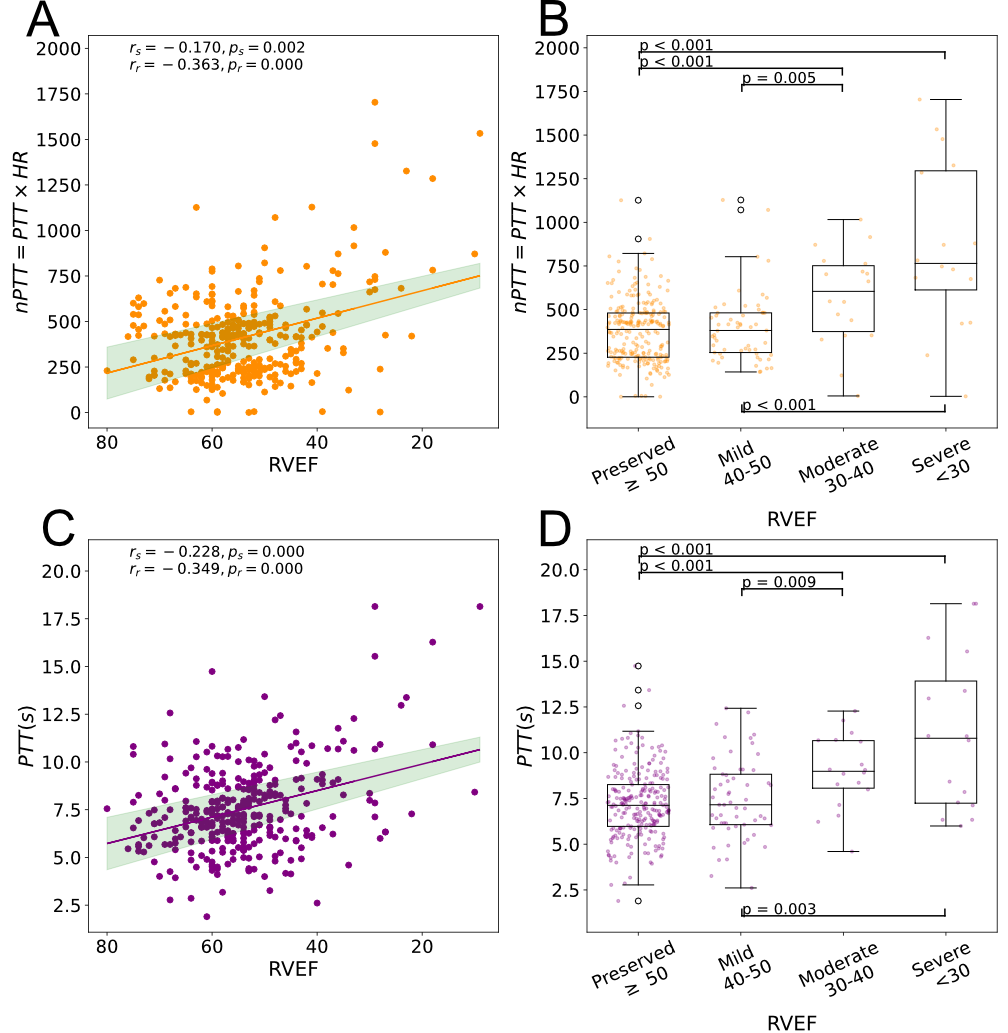


*Supplement Figure 5S*: A) Correlation between normalized pulmonary transit time (nPTT) and right ventricular ejection fraction (RVEF); B) nPTT according to groups of RVEF; C) Correlation between pulmonary transit time (PTT) and RVEF; D) PTT according to groups of RVEF.


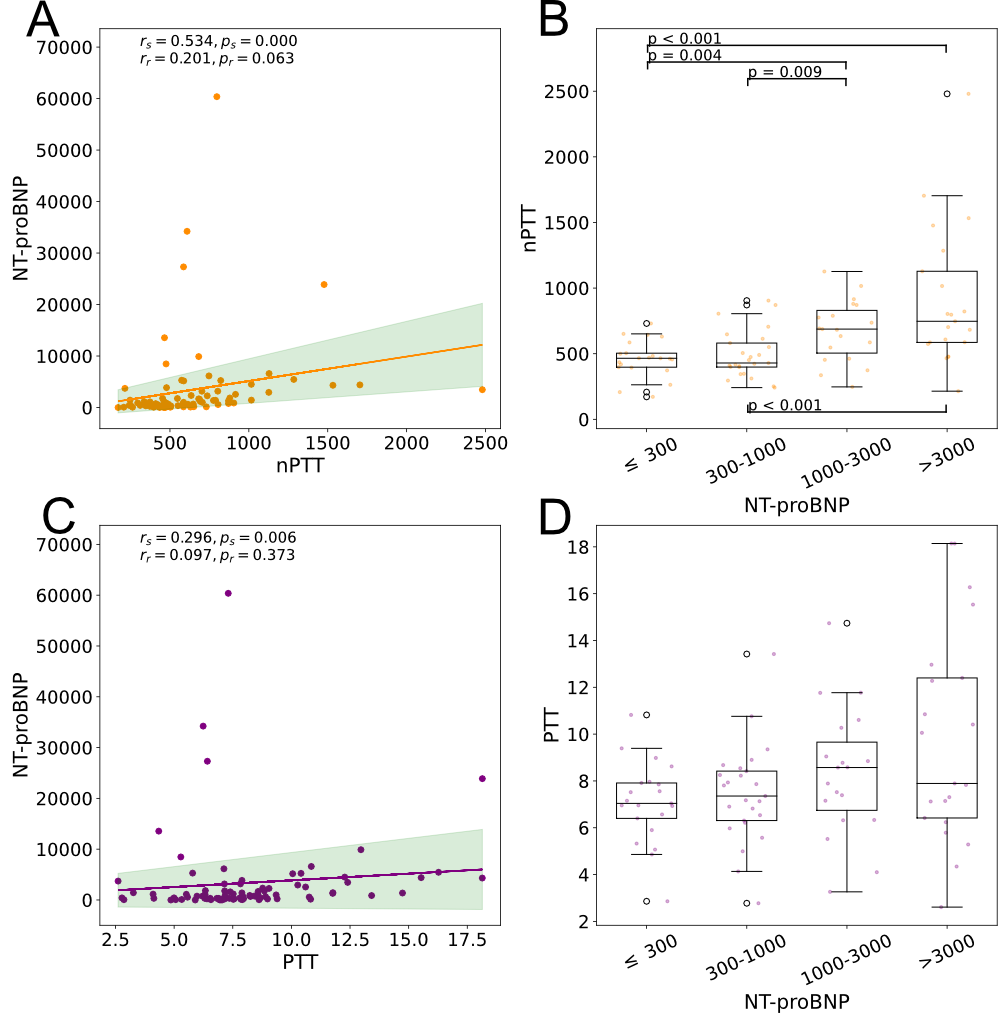


*Supplement Figure 6S*: A) Correlation between normalized pulmonary transit time (nPTT) and N-terminal pro B-type natriuretic peptide (NT-proBNP); B) nPTT according to groups of NT-proBNP; C) Correlation between pulmonary transit time (PTT) and NT-proBNP; D) PTT according to groups of NT-proBNP.


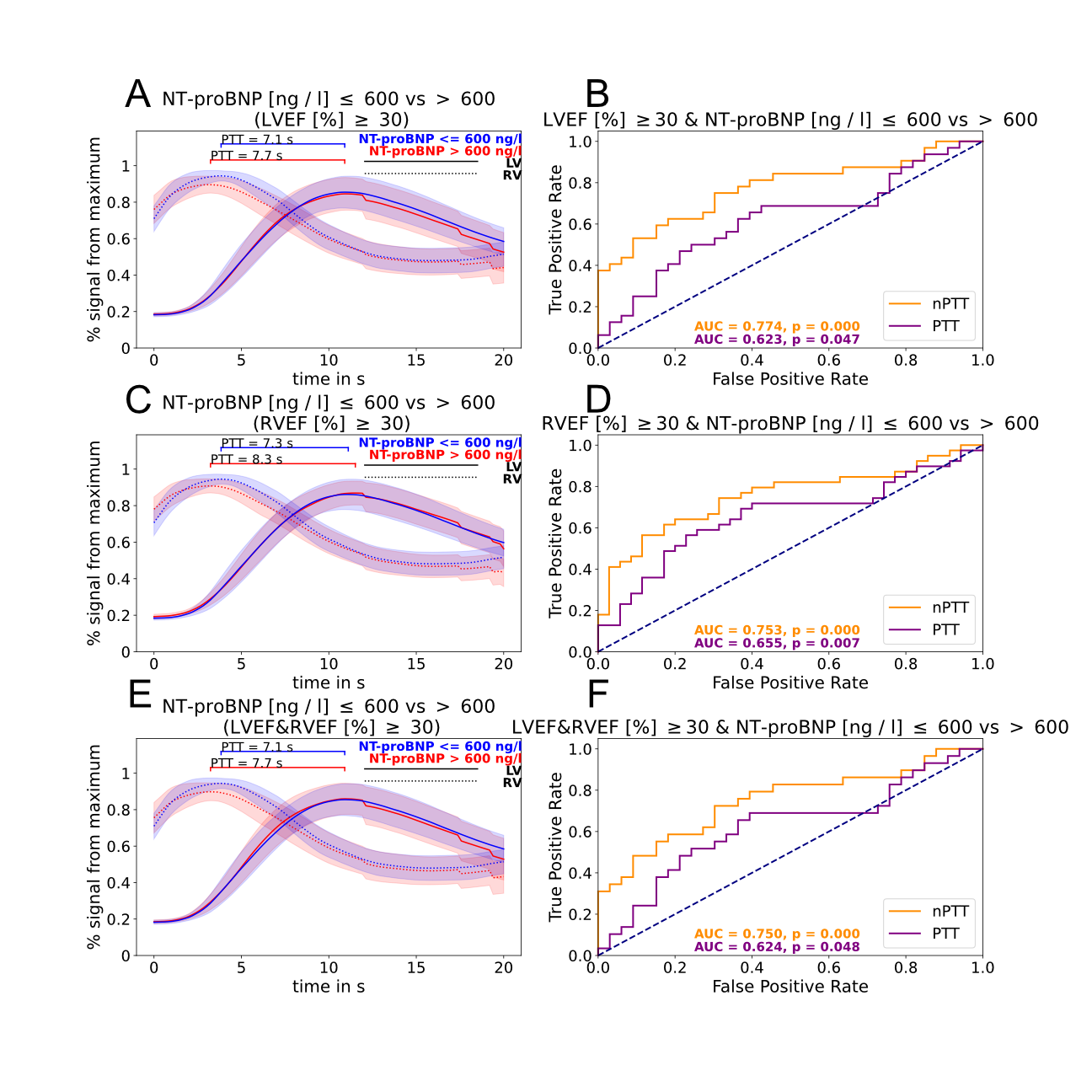


*Supplement Figure 7S*: A) Values of pulmonary transit times (PTT) of patients with left ventricular ejection fraction greater than or equal to 30% (LVEF >=30%; n= 65) and with NT-proBNP <= and > 600 ng/L respectively with NT-proBNP <= and > 300 ng/L for patients with BMI > 35 kg/m^2^; B) diagnostic performance of PTT and nPTT in patients with LVEF greater than or equal to 30% (>=30%) in receiver operating characteristic (ROC) curve analysis for the inclusion of heart failure; C) Values of PTT of patients with right ventricular ejection fraction greater than or equal to 30% (RVEF >=30%; n= 74) and with NT-proBNP <= and > 600 ng/L respectively with NT-proBNP <= and > 300 ng/L for patients with BMI > 35 kg/m^2^; D) diagnostic performance of PTT and nPTT in patients with RVEF greater than or equal to 30% (>=30%) in ROC curve analysis for the inclusion of heart; E) Values of PTT of patients with LVEF and RVEF greater than or equal to 30% (both >=30%; n= 62) and with NT-proBNP <= and > 600 ng/L respectively with NT-proBNP <= and > 300 ng/L for patients with BMI > 35 kg/m^2^; F) diagnostic performance of PTT and nPTT in patients with both LVEF and RVEF greater than or equal to 30% (>=30%) in ROC curve analysis for the inclusion of heart failure. Inclusion of heart failure was defined as NT-proBNP <= and > 600 ng/L respectively with NT-proBNP <= and > 300 ng/L for patients with BMI > 35 kg/m^2^.
